# Effects of applying a standardized assessment and evaluation protocol in housing adaptation implementation – results from a quasi-experimental study

**DOI:** 10.1186/s12889-019-7815-9

**Published:** 2019-11-04

**Authors:** A. Malmgren Fänge, G. Carlsson, A. Axmon, B. Thordardottir, C. Chiatti, M. H. Nilsson, L. Ekstam

**Affiliations:** 1 Department of Health Sciences, Lund University, SE-221 00 Lund, Sweden; 2 Division of Occupational and Environmental Medicine, EPI@LUND (Epidemiology, Population studies, and Infrastructures at Lund University), Lund University, SE-221 00 Lund, Sweden; 3 Faculty of Health Sciences, OsloMet - OsloMetropolitan University, NO-0130, Oslo, Norway; 4 Department of Clinical Sciences, Lund University, Lund, Sweden

**Keywords:** Home modification, Evaluation, Disability, Activities of daily living, Usability, Health related quality of life, Participation, Complex intervention, Occupational therapy

## Abstract

**Background:**

Standardized, research-based strategies to guide the implementation and evaluate the effects of housing adaptations (HA) on client outcomes are rare. We hypothesized that, compared to ordinary practice, a standardized assessment and evaluation protocol for HA implementation would better maintain or improve client outcomes over 1 year.

**Method:**

Using a cluster design, South Swedish municipalities were recruited to an intervention or control group. Data on activities of daily living, usability of the home, health related quality of life, and participation frequency and satisfaction were collected at home visits 1 month before the HA (baseline; T1), and at 3 (T2), 6 (T3) and 12 (T4) months after. In the intervention group (*n* = 112) data were collected according to a standardized protocol while in the control group (*n* = 129) ordinary routines were applied. Changes from baseline to subsequent time points were categorized as no deterioration (i.e. improvement or no change) or deterioration, for each outcome item separately. Differences in “no deterioration” between the groups were assessed using logistic regression.

**Results:**

Little effect of using the standardized protocol was detected. For activities of daily living, statistically significant differences between the groups were found for toileting (T1-T4; OR 3.14), dressing (T1-T4; OR2.89) and cooking (T1-T3 and T1-T4; OR 3.14). For usability of the home differences were found in personal hygiene (T1-T2; OR 2.32) using a wheelchair (T1-T2 and T1-T3; OR 9.50), picking up the mail (T1-T3; OR 4.06), and in participation, helping others (T1-T3 and T1-T4; OR 2.33 and 3.36).

**Conclusion:**

The applied standardized protocol for HA implementation did not show any convincing effect, possibly due to the complexity of the intervention itself, and the implementation process. A process evaluation might generate in-depth knowledge about the reasons behind the findings.

**Trial registration:**

ClinicalTrials.gov. NCT01960582.

## Background

Over a billion people are estimated to live with some form of disability, and among them, between 110 (2.2%) and 190 million (3.8%) people aged 15 years and older have significant difficulties in functioning [1]. The proportion of people with disability is increasing, in part due to ageing populations and an increase in chronic health conditions [2].

Being active and participating in everyday life despite increasing age and disability is considered to be crucial for health and well-being [1]. Many interventions that aim at improving activity and participation focus on aspects such as training and the provision of mobility devices, but the influences of the built environment are also considerable [3]. Among older people and people with disabilities in particular, the home environment plays a crucial role in enhancing everyday life. Thus, different interventions in the home requires special attention in order to compensate for reduced functional capacity, and support activity and participation [4]. Housing adaptation (HA) is one intervention that provides solutions on a case-by-case basis to meet the specific needs of a person, that is, to enhance independent living in the own home [5].

HA denotes changes to the physical environment, such as the removal of features or the installation of new ones. Each adaptation should be tailored to the individual needs of each client and may thus vary in extent, from removal of single thresholds to full renovations of e.g. bathrooms and kitchen [5, 6]. Home modifications, a related but broader concept, include HA as well as adaptations such as rearrangement of furniture and provision of assistive technology and assistive devices [7]. In Sweden, the full costs of a HA can be covered by the municipality after application by the client [3]. A certificate (issued by a health professional) that states the need of the intervention has to be attached to the application.

The population receiving HA is very heterogeneous regarding aspects of health as well as standards and type of housing they live in. In Sweden, 72% of the people receiving HA are older than 70 years [6] and the majority is facing age-related health decline and dependence (see, for example [7, 8]). However, younger or middle-aged people with acute or progressive diseases or injuries are also HA recipients [9]. The majority of HA clients receive health care and social services interventions in parallel, such as provision of mobility devices and assistance with activities of daily living, ADL (see e.g. [9–12]).

Comparing studies on HAs is problematic, since different definitions of what constitutes a HA are applied, including the use of different variables and methods to measure outcomes [8, 11], as well as different time spans between follow-ups [8]. It has been demonstrated that HAs improve activity performance and reduce dependence on other people [13–19], and the usability of the home [13, 14], wellbeing [20] and participation [21, 22]. However, what would be the ideal follow-up times to detect clinically important information has previously not been investigated.

The current Swedish HA regulation [5] provides no details on how to assess the needs of the client and extents of the HA required, and guidance for practice is lacking. In practice contexts, systematic approaches to HA delivery are used to some extent [23]. It is known that health care and social services interventions that include structured assessments by specifically trained staff are more effective than interventions based on non-structured assessments (see e.g. [23, 24]). Despite this, the majority of HA are implemented by applying a professional judgement without a structured methodology to all steps in the process and without the use of current research evidence on which outcomes to evaluate [25, 26]. Further development and evaluation of existing interventions that enhance functioning and independent living in one’s own home is needed. However, standardized assessment and evaluation protocols based on research findings, to guide the HA implementation process are lacking, and studies have rarely focused on the outcomes of the use of such protocols on HA. Accordingly, in this study we hypothesized that, compared to ordinary practice, applying a systematic, standardized assessment and evaluation protocol on the implementation of HA would lead to a larger increase in the usability of the home and to unchanged or larger increase in independence in ADL, participation frequency and satisfaction, and health-related quality of life at different time-points over 1 year.

The research question is as follows:

Are there any differences in changes between baseline and 3, 6 and 12 months respectively of applying a standardized assessment and evaluation protocol for HA implementation compared to unstructured assessment and evaluation procedures on ADL, usability of the home, participation and health related quality of life?

## Methods

### Trial design

This study is part of a quasi-experimental, cluster design trial, the Research Strategy for Housing Adaptation (ResHA) trial, applying a before–after design [27]. South Swedish municipalities were recruited based on a cluster design, that is, the entire municipalities were recruited as intervention or control sites. At all sites the clients received HA if they were judged needed by an occupational therapist, however, the procedures for implementation differed between intervention and control sites. Identical data collection was performed at the same four time points: at T1 (max. 1 month before the start of the HA) and 3, 6, and 12 months after the HA was finalized (T2, T3, and T4, respectively), using the same assessment instruments. Owing to the nature of the intervention and the study design, there was no blinding to group assignment. This applies to the study participants, those administering the interventions, and the assessors.

### Settings

Three medium-sized municipalities (approximately 40,000–50,000 inhabitants) in the south of Sweden were included. Because of the project’s complexity, duration and the effort required for data collection, the staff members and the management needed to express a sincere interest to partake in the study in order for the municipality to be enrolled. In addition, a readiness to change their practices was a prerequisite to become an intervention municipality. Two of the municipalities accepted to become intervention sites and one municipality accepted to become a control site. Before the study started, there was a variation in the number of accepted HA applications granted in the three municipalities (between 3.4 and 10.5 per 1000 inhabitants, i.e., around 137–446 per year in each municipality) [6], the higher number representing the control site.

### Participants

All persons above 20 years of age living in ordinary housing and who applied for a HA grant, via the occupational therapists (n = approximately 45) employed by any of the three municipalities, were considered eligible to participate in the study. Exclusion criteria were living in sheltered housing and an inability to communicate or follow instructions in Swedish. All municipalities used the same inclusion and exclusion criteria.

### Intervention

The intervention for this study consisted of the use of a standardized, structured assessment and evaluation protocol for HA implementation. The intervention was developed based on earlier research and current legislative frameworks for HA implementation in Sweden, for details see [27]. At the intervention sites, occupational therapists applied their usual professional judgements skills, but they also applied the protocol. The intervention guided the occupational therapists with standardized procedures for the assessment and evaluation of person-, activity-, and housing-related aspects, i.e. the primary and secondary outcomes, at home visits before the HA, and 3, 6 and 12 months after the HA was finalized. Each study participant was assessed by the occupational therapist responsible for their HA.

Prior to the start of the data collection, the occupational therapists attended an extensive training course targeting the rationale for applying the structured assessment and evaluation protocol, assessment procedures, issues of validity and reliability related to instruments, procedures and results, as well as basic statistics, and consequences of high attrition rates and low protocol adherence. The occupational therapists conducted test assessments for inter-rater reliability purposes. During the data collection period (2014–2017) the project managers visited the interventions sites frequently and were at hand over the telephone.

### Control

At the control site, the occupational therapists worked according to their ordinary practice routines for HA implementation. This included collecting information of importance (based on experience and ordinary practice routines) to be able to write a certificate concerning the need to receive a HA grant. For some clients, follow-ups after the HA were performed. However, there was no clear structure with respect to client characteristics or assessments applied for these (see also [26]). At the control site, the occupational therapists did not have access to the data collected for the study. Instead, data were collected by a trained occupational therapist employed for the project.

In all sites, the occupational therapists tailored each client’s HA based on the different evaluation results, independently of whether they belonged to the intervention or control municipalities.

### Outcomes

Primary outcomes were activities of daily living (ADL) and usability of the home. Secondary outcomes were participation frequency and satisfaction, and health related quality of life. The outcomes were selected based on current Swedish HA legislation with its aim of enhancing independent living in the own home [5], in our study operationalized as ADL dependence and participation frequency and satisfaction. Moreover, given the close relationship of the HA legislation to current Swedish planning and building legislation [5, 27, 28], usability of the home is a key outcome for environmental interventions, in particular since it relates to the design of the environment with the possibility to perform activities [13, 14]. Furthermore, health related quality of life is an ultimate goal of all health related interventions and thus included in this study.

#### Activities of daily living

Dependence and difficulty in ADL, measured by the ADL Staircase was used as a primary outcome. The ADL staircase comprises nine items on feeding, transfer, using the toilet, dressing, bathing, cooking, transportation, shopping, and cleaning. Originally, the following response categories were applied: “independent” “partly dependent”, and “dependent”, with dependent/independent denoting the need of help from another person to perform the activity [29]. However, recent research has highlighted the usefulness of more precise information of whether activities are performed with or without difficulty (see e.g. [30]). Therefore, in the present study, each item included an amendment that clarified whether the person was independent without or with difficulties.

#### Usability of the home

Usability of the home was measured by a revised version of Usability in My Home (UIMH) instrument [31, 32]. Usability of the home denotes the effectiveness of, efficiency of, and client satisfaction with the home environment. It focuses on the performance of tasks and activities and the related perception of satisfaction [33]. The instrument comprises self-reported 18 items reflecting the respondent’s satisfaction with the home environment in relation to performance of different personal, instrumental, leisure, and socially related activities. The response alternatives range from 1 to 5, higher scores imply higher perceived usability of the home.

#### Participation frequency and participation satisfaction

Participation was assessed by means of study-specific questions based on previous research [34] and on the goals of HA as expressed in the legislation [5]. Each client responds to eight statements in relation to how often (frequency) and how satisfied (satisfaction) the client was with participation in relation to 1) having contacts with others in your home, 2) helping others, 3) doing something outside the home with others, and 4) doing something outside the home alone. The response alternatives range from 1 to 5, higher scores imply higher frequency and satisfaction, respectively. Data were analyzed item-wise.

#### Health related quality of life

Data regarding health related quality of life were collected using the EQ-5D and assessed using each item of the EQ-5D-5 L as well as the EQ index and EQ VAS separately [35]. The EQ-5D-5 L addresses five dimensions of health, namely mobility, self-care, usual activities, pain/discomfort and anxiety/depression on a five-graded ordinal scale. The response alternatives range from 1 to 5, where 1 indicates no and 5 severe difficulties. The respondent’s scoring obtained on these dimensions can be converted to a single summary index number reflecting preferability compared to other health profiles. This EQ index ranges from 1 (perfect health) through 0 (death), to minus 0.59 (worse than death). As there is no reference population for Swedish data, the Danish reference population for EQ-5D-5 L was used to assign an EQ index to each person at each time point [34].

Respondents are also asked to rate their overall health on the day of the interview on the vertical visual analogue scale, EQ VAS, from 0 to 100, where 0 indicates the worst and 100 the best imaginable health. The EQ-5D has been tested for validity and reliability [35, 36].

#### Descriptive data

Information about age, sex, educational level, living conditions, housing standards and civil status was registered based on self-reported data, and cognitive functioning was assessed using the Montreal Cognitive Assessment scale, MoCA [37]. See Table 1 for sample description.

**Table 1:** Sample description at baseline (T1)

	Control site (*n* = 129)		Intervention sites (*n* = 112)		p
	n	%	n	%	
Gender					
Men	47	36.4%	42	37.5%	0.864
Women	82	63.6%	70	62.5%	
Age					
≤ 64	24	17.8%	17	15.2%	0.234
65–74	19	14.7%	27	24.1%	
75–84	51	39.5%	45	40.2%	
≥ 85	33	25.6%	21	18.8%	
missing	3	2.3%	2	1.8%	
Living arrangements					
living alone	71	55.0%	63	56.3%	0.615
living with others	58	45.0%	48	42.9%	
missing	0	0%	1	0.9%	
Education					
Primary School	87	67.4	67	59.8	0.122
High School	19	14.7	29	25.9	
University or higher	16	12.4	15	13.4	
missing	7	5.4	1	0.9	
Cognitive impairment^a^					
27–30	22	17.1%	20	17.9%	0.329
18–26	47	36.4%	72	64.3%	
10–17	13	10.1%	15	13.4%	
Missing	47	36.4%	5	4.5%	

^a^ Measured using the Montreal Cognitive Assessment, MoCA (37);

### Data analysis

Potential differences between the two groups at baseline (T1) were evaluated using the Mann-Whitney U-test (ordinal data, i.e. items within ADL Staircase, EQ-5D-5 L, UIMH, participation frequency and satisfaction) or ANOVA (continuous data, i.e. EQ index and EQ VAS).

Change from T1 to subsequent time points in the intervention group was compared with the corresponding changes in the control group. This was done for each outcome variable separately. When analyzing item-data, change from baseline (T1) was categorized as **no deterioration** (i.e. having the same or a better score than at T1) or **deterioration** (i.e. having a worse score than at T1). For each analysis logistic regression was used to estimate odds ratios (ORs) with 95% confidence intervals (CIs).

The distributions of the EQ index and the EQ VAS were assessed using P-P-plots and were considered normally distributed. The same was true for the changes in EQ index and EQ VAS. Thus, potential differences between the groups were assessed using analysis of variance (ANOVA).

## Results

### Participant flow

**Fig. 1:**
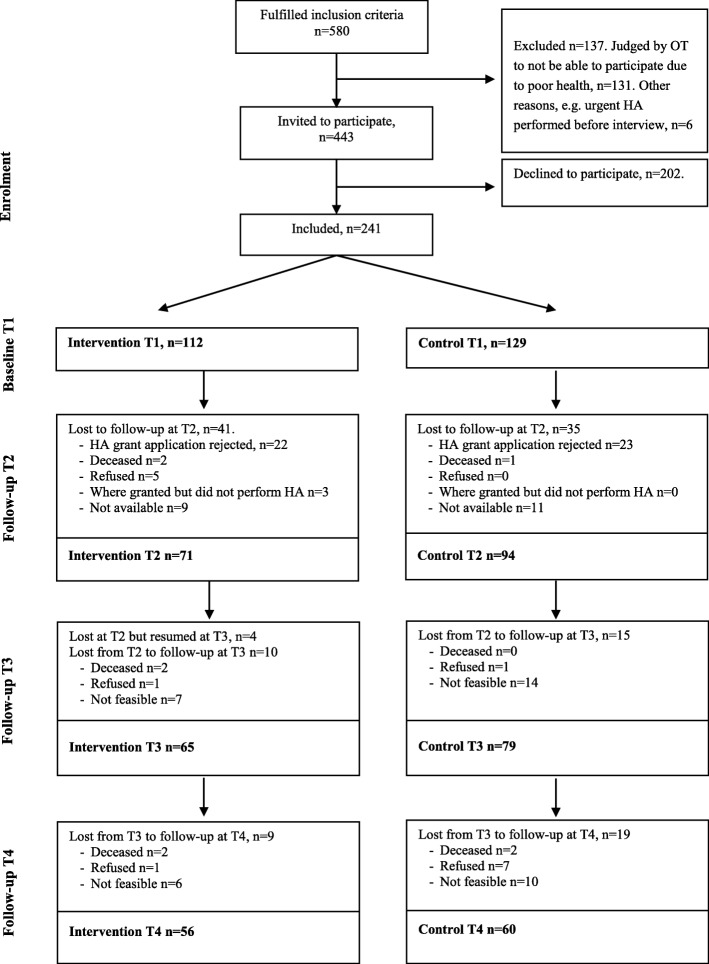
Participant flow chart

In total, a consecutive sample of 580 persons met the inclusion criteria, but 131 of these were judged by the occupational therapists as unable to participate due to poor health. Six additional persons were excluded due to other reasons, e.g. that the HA was urgent and performed before the first interview could take place. The remaining 443 individuals were invited to participate, but 202 (46%) declined, adding up to 241 persons that accepted to participate in the study. However, 45 of them had their HA application turned down and was therefore only included at baseline. That is, the final study sample consisted of 241 clients at baseline (intervention (I): *n* = 112, control (C): *n* = 129), 165 after 3 months (I: *n* = 71; C: *n* = 94), 144 after 6 months (I: *n* = 65; C: *n* = 79) and 116 after 12 months (I: *n* = 56; C: *n* = 60). See Fig. 1.

### Baseline data

No differences in the basic descriptive statistics was found between the intervention and the control group (Table 1). The intervention group had higher scores for independence in ADL/feeding (*p* < 0.001, Table 2), but no other statistically significant differences in ADL were found between the two groups (data not shown). For usability of the home, the intervention group had lower scores for picking up the mail (*p* = 0.018) (Table 3), compared to the control group, but no statistically significant differences were found for any of the other items (data not shown). Participation frequency was lower in the intervention group for contact with others (in the home) (*p* = 0.037) and doing activities outside the home alone (*p* = 0.014; Table 3). There were no other statistically significant differences for participation frequency, nor for any of the participation satisfaction items (data not shown). For health related quality of life, the intervention group had lower scores for mobility (*p* < 0.001) and pain and discomfort (*p* = 0.016; Table 3). The mean EQ index was 0.58 (SD 0.26) in the control group versus 0.50 (0.25) in the intervention group, *p* = 0.009. The corresponding values for the EQ VAS were 60 (21) and 53 (20), *p* = 0.011. No other statistically significant differences at baseline were found for any of the variables describing health related quality of life.

**Table 2:** Sample description [%]: ADL at baseline (T1)

	Indep wo diff		Indep w diff		Partly dep		Dependent	
Instrument / Variable	Control	Intervention	Control	Intervention	Control	Intervention	Control	Intervention
*ADL Staircase*								
Feeding^b^	49%	82%	43%	8%	8%	8%	0%	2%
Transfer^b^	31%	47%	65%	41%	4%	7%	0%	5%
Toileting^b^	47%	62%	46%	26%	6%	5%	1%	6%
Dressing^b^	30%	40%	47%	24%	11%	19%	12%	17%
Bathing^c^	23%	30%	42%	25%	18%	22%	17%	24%
Cooking^b^	25%	39%	40%	17%	9%	16%	26%	28%
Transportation^a^	18%	23%	20%	13%	53%	46%	9%	19%
Shopping	15%	16%	19%	13%	26%	29%	40%	42%
Cleaning	5%	6%	19%	5%	6%	14%	70%	74%

^a^ 5 missing values in the control group; 1 missing values in the intervention group

^b^ 1 missing values in the intervention group

^c^ 2 missing values in the intervention group

**Table 3:** Sample description [n, mean and SD]: Usability in My Home, participation and EQ-5D at baseline (T1)

	Control			Intervention		
Instrument / Variable	n	Mean	(SD)	n	Mean	(SD)
Usability in My Home						
Use the toilet	127	3.90	1.52	109	3.89	1.42
Personal hygiene	127	3.83	1.37	109	3.64	1.62
Prepare meals	105	3.93	1.07	84	3.94	1.15
Prepare snacks	112	4.21	1.00	94	4.11	0.80
Move around with or without mobility device	115	3.67	1.56	101	3.65	1.41
Use a wheelchair	22	3.55	1.98	41	3.07	1.38
Wash by hand	41	3.85	1.10	44	3.50	1.39
Use the washing machine	67	4.01	1.12	62	3.77	1.50
Light cleaning	70	3.66	1.28	66	3.62	1.14
Vacuum/clean the floors	41	3.41	1.80	39	3.28	2.00
Manage garbage	72	3.53	1.69	62	3.34	1.68
Enter/leave the home	123	3.22	1.94	104	2.99	1.82
Pick up the mail	90	4.03	1.25	81	3.65	1.36
Engaging in hobbies/leisure at home	69	4.03	1.10	76	3.74	1.35
Work/study at home	6	3.50	1.58	24	3.63	1.40
Socialize/care for family/friends	110	4.14	1.01	102	4.06	0.78
Contact others by phone/computer	122	4.16	1.10	104	4.29	0.78
Use TV/radio	124	4.21	1.00	107	4.47	0.59
Participation frequency						
In the home	129	4.35	0.66	109	4.07	1.06
Helping others	128	1.80	1.59	98	1.71	1.33
Outside the home with others	128	3.00	1.86	108	2.81	1.43
Outside the home alone	127	2.97	2.60	106	2.48	2.04
Participation satisfaction						
In the home	128	4.07	1.33	109	4.06	0.96
Helping others	124	3.09	1.87	94	3.05	1.39
Outside the home with others	128	3.37	1.89	107	3.49	1.28
Outside the home alone	125	3.28	1.90	103	2.99	1.29
EQ-5D						
Mobility	128	2.59	1.38	109	3.37	1.19
Self-care	129	2.20	1.26	109	2.34	1.33
Usual activities	128	2.81	1.82	108	3.02	1.63
Pain/discomfort	128	2.72	1.36	108	3.08	0.84
Anxiety/depression	128	1.91	1.15	109	1.93	0.82

### Outcomes

Overall, only a few significant differences in changes in outcomes between the different time points were found between intervention and control group.

#### Activities of daily living

**Fig. 2:**
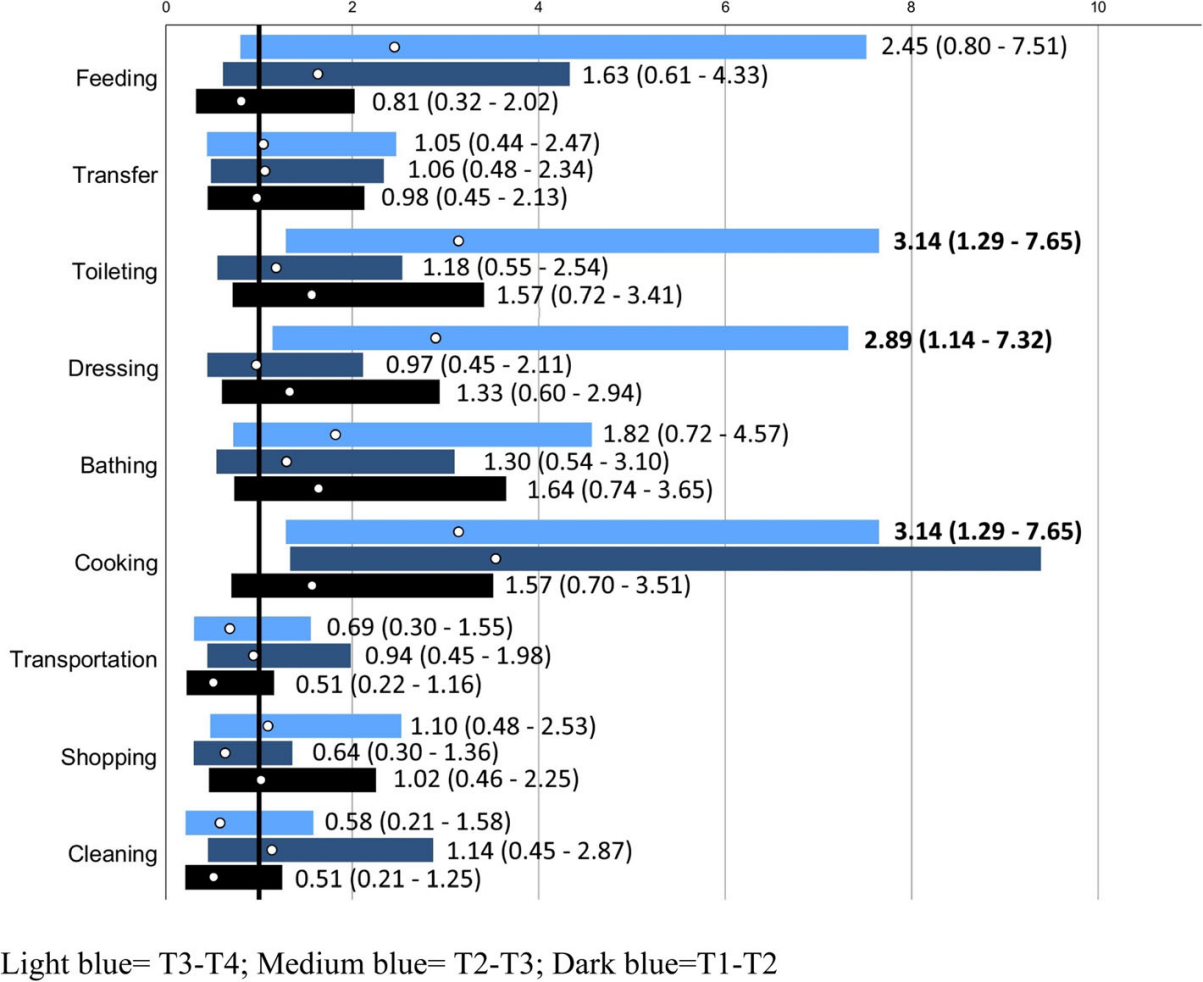
Odds Ratios (ORs; white circles) with 95% confidence interval (bars; dark blue for changes T1-T2, medium blue for changes T1-T3 and light blue for changes T1-T4 for people in the intervention group vs control regarding “no deterioration in ADL”. The solid black line marks OR = 1, i.e. no differences between groups

People in the intervention group were more likely to not deteriorate in independence in cooking between T1 and T3 and T4 respectively, and also more likely to not deteriorate in independence in toileting and dressing between T1 and T4 (Table 4, Fig. 2).

**Table 4:** Percentage of people with “no deterioration” for control and intervention group at each time-point (T2, T3 and T4) during follow-up, in relation to in−/dependence in ADL, usability of the home, participation and health related quality of life at baseline (T1)

	T2		T3		T4	
Instrument / Variable	Control	Intervention	Control	Intervention	Control	Intervention
ADL Staircase						
Feeding	88%	86%	82%	88%	79%	90%
Transfer	81%	80%	76%	77%	74%	75%
Toileting	76%	83%	72%	75%	60%	83%
Dressing	79%	83%	76%	75%	66%	85%
Bathing	77%	84%	80%	84%	72%	83%
Cooking	78%	85%	72%	90%	60%	83%
Transportation	87%	77%	70%	69%	70%	62%
Shopping	81%	81%	77%	68%	71%	73%
Cleaning	89%	81%	84%	85%	86%	78%
Usability in My Home						
Use the toilet	76%	76%	78%	83%	64%	76%
Personal hygiene	70%	85%	77%	75%	64%	80%
Prepare meals	75%	77%	69%	78%	74%	86%
Prepare snacks	69%	84%	67%	78%	65%	79%
Move around with or without mobility device	76%	79%	80%	72%	74%	84%
Use a wheelchair	50%	90%	50%	90%	33%	76%
Wash by hand	75%	67%	92%	89%	100%	83%
Use the washing machine	79%	93%	81%	78%	68%	85%
Light cleaning	67%	85%	79%	86%	69%	79%
Vacuum/clean the floors	62%	83%	64%	80%	25%	80%
Manage garbage	69%	71%	76%	76%	67%	89%
Enter/leave the home	78%	89%	84%	90%	77%	89%
Pick up the mail	69%	88%	71%	91%	69%	82%
Engaging in hobbies/leisure at home	79%	78%	69%	79%	60%	76%
Work/study at home						
Socialize/care for family/friends	74%	74%	76%	94%	77%	89%
Contact others by phone/computer	74%	81%	58%	78%	65%	70%
Use TV/radio	75%	88%	67%	78%	66%	70%
Participation frequency						
In the home	89%	77%	90%	83%	86%	73%
Helping others	83%	81%	77%	81%	75%	70%
Outside the home with others	78%	80%	75%	80%	68%	78%
Outside the home alone	84%	86%	86%	76%	86%	75%
Participation satisfaction						
In the home	72%	82%	77%	80%	78%	78%
Helping others	69%	68%	60%	78%	53%	79%
Outside the home with others	76%	73%	68%	79%	68%	80%
Outside the home alone	71%	76%	83%	80%	78%	77%
EQ-5D						
Mobility	63%	76%	79%	87%	73%	78%
Self-care	74%	77%	71%	76%	59%	72%
Usual activities	76%	73%	74%	80%	64%	76%
Pain/discomfort	74%	82%	75%	85%	78%	76%
Anxiety/depression	76%	80%	78%	87%	75%	78%

#### Usability of the home

**Fig. 3:**
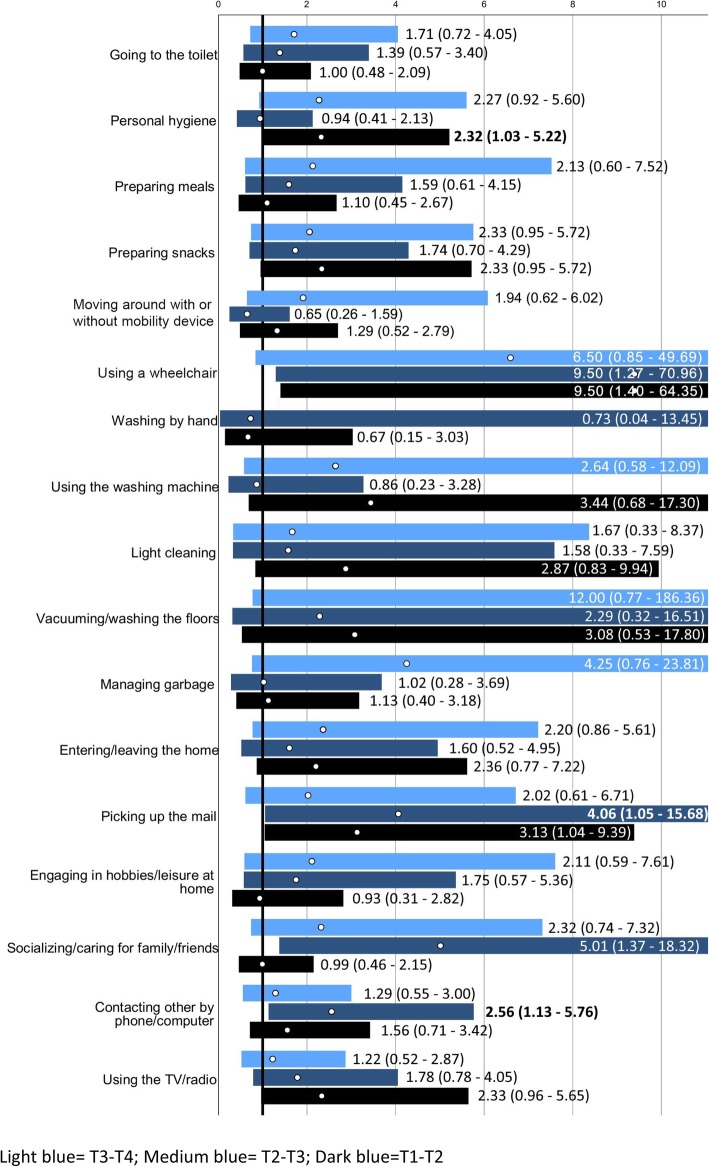
Odds Ratios (ORs; white circles) with 95% confidence interval (bars; dark blue for changes T1-T2, medium blue for changes T1-T3 and light blue for changes T1-T4 for people in the intervention group vs control regarding “no deterioration in Usability of the home”. The solid black line marks OR = 1, i.e. no differences between groups

People in the intervention group were more likely to not deteriorate in independence in personal hygiene between T1 and T2, but this effect did not remain at T3 or T4 (Table 4, Fig. 3). The usability of the home was also more likely to not deteriorate for picking up the mail between T1 and T2 and T3 respectively, but not between T1 and T4, and for socializing/caring for family/friends and contacting other by phone/computer between T1 and T3, but not between T1 and the other time point.

#### Participation frequency and participation satisfaction

**Fig. 4:**
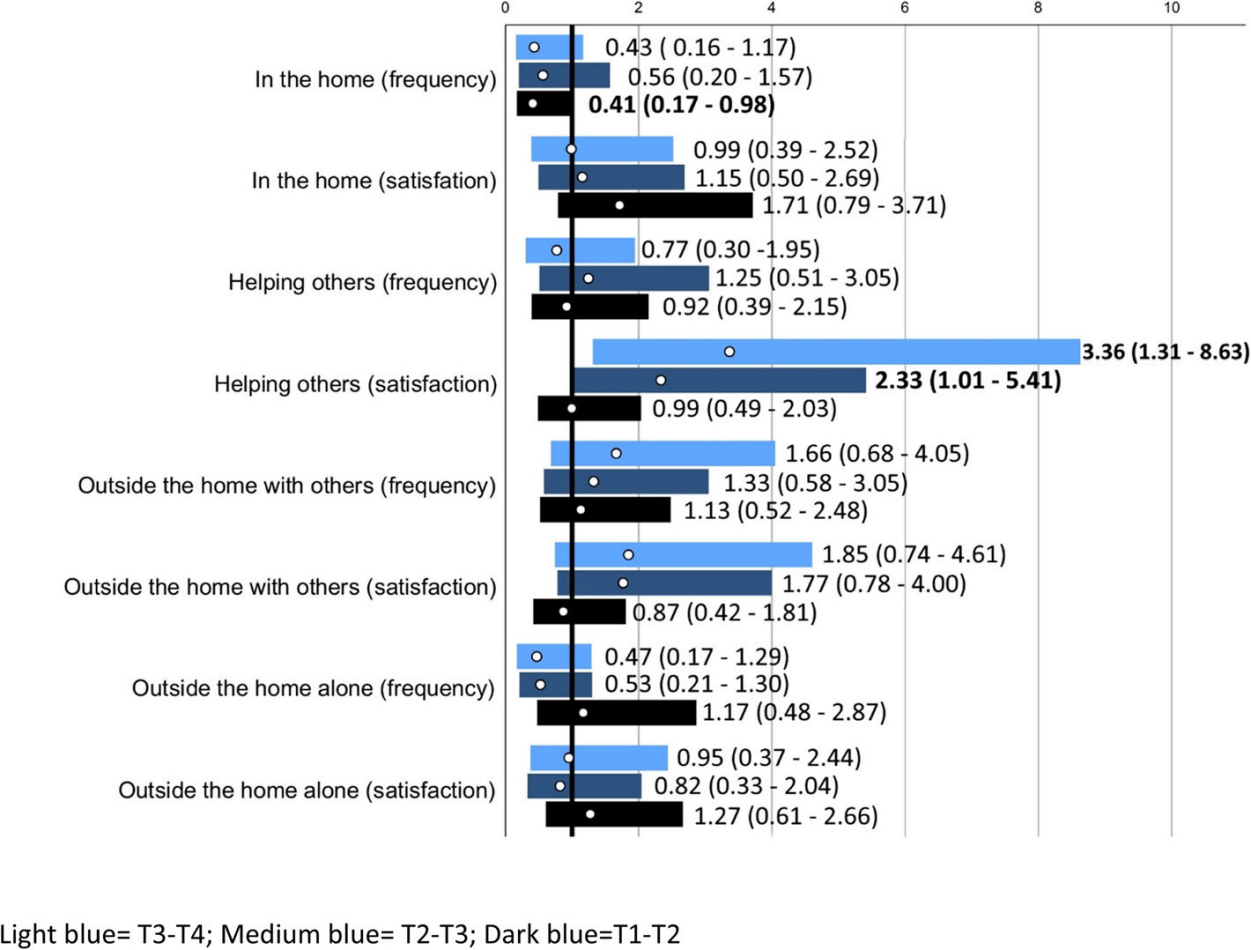
Odds Ratios (ORs; white circles) with 95% confidence interval (bars; dark blue for changes T1-T2, medium blue for changes T1-T3 and light blue for changes T1-T4 for people in the intervention group vs control regarding “no deterioration in participation”. The solid black line marks OR = 1, i.e. no differences between groups

People in the intervention group were less likely to not deteriorate in participation frequency in the home between T1 and T2, but not between T1 and T3 or T4 respectively (Table 4, Fig. 4). They were more likely to have an increased satisfaction in helping others between T1 T3 and T4, but not between T1 and T2.

#### Health related quality of life

**Fig. 5:**
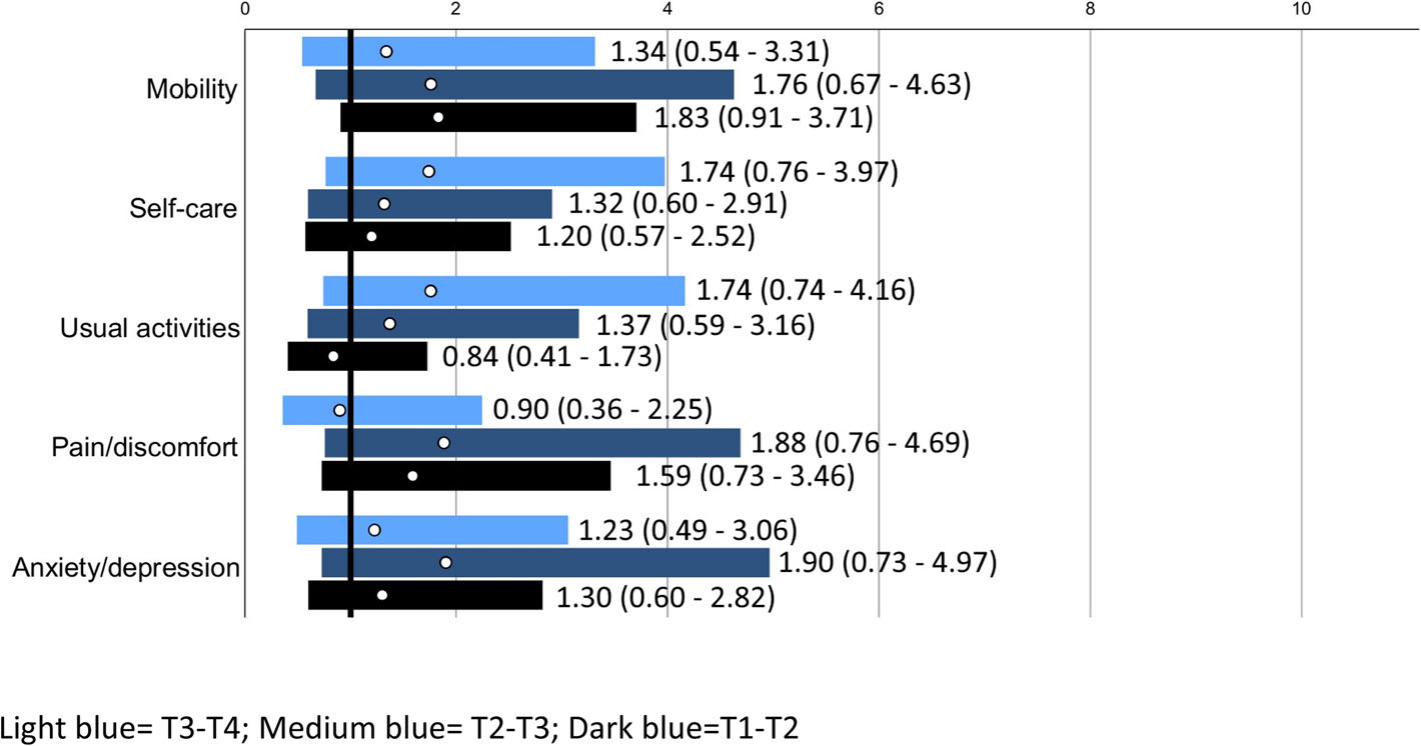
Odds Ratios (ORs; white circles) with 95% confidence interval (bars; dark blue for changes T1-T2, medium blue for changes T1-T3 and light blue for changes T1-T4 for people in the intervention group vs control regarding “no deterioration in health related quality of life”. The solid black line marks OR = 1, i.e. no differences between groups

There were no differences between the two groups for any of the items of the EQ-5D-5 L at any of the time points (Table 4, Fig. 5). Furthermore, the two groups did not differ (*p* = 0.92) in EQ index regarding change from T1 to T2: mean change in controls was 0.02 (SD 0.28) vs. 0.02 (SD 0.17) in the intervention group. This applied also from T1 to T3 (controls − 0.05 (0.25) vs. intervention − 0.03 (0.22), *p* = 0.62), and from T1 to T4 (controls 0.00 (0.23) vs. intervention 0.01 (0.27); *p* = 0.77). Moreover, no statistically significant differences were found for EQ VAS from T1 to T2 (controls − 1.79 (23.78) vs. intervention − 4.06 (19.21); *p* = 0.53), from T1 to T3 (controls − 4.06 (26.19) vs. intervention 7.85 (19.30); *p* = 0.38), or from T1 to T4 (controls − 2.16 (26.31) vs. intervention 0.48 (23.77; *p* = 0.61).

## Discussion

We investigated whether applying a standardized assessment and evaluation protocol for HA implementation had an effect on changes in independence in ADL, usability of the home, participation frequency and satisfaction, as well as in health related quality of life, compared to ordinary occupational therapy practice in relation to HA. Our hypothesis was that applying a standardized assessment and evaluation protocol to HA implementation would be more effective than HA implemented based on ordinary occupational therapy practice only. That is, we assumed that by applying a structured protocol the occupational therapists would gain more in-depth information about the client’s needs and thus be able to tailor the HA to be more client-centered [4, 38, 39] and activity-based [39]. Thus, we expected differences in changes over time between intervention and control groups. However, the significant differences in changes found seem to be more of a random character, and our hypothesis could therefore not be confirmed. This means that the potential effects of the intervention on the occupational therapist’ professional judgments and clinical reasoning as a basis for HA implementation did not have an impact on the outcomes for the client. Instead, recent studies for example highlight the need for an even more client-centered approach to HA, i.e. to contribute with more than just structural adaptations, since even minor repairs may be vital for older adults [40, 41].

A HA can be considered as a complex intervention [42–45], given the number of interacting components related to the person, the housing environment and the activities to be performed in the home. The target intervention of our study strived for standardization [38], i.e. that all HA clients should receive the same assessments and evaluations at the same time points independently of their problems, needs and goals [27]. The rationale for choosing the primary and secondary outcomes as well as the standardized follow-up scheme applied in this study was based on research, documents and current Swedish HA as well as planning and building legislation. However, despite the thorough work behind this choice, this might not have been the best methodology for evaluation of the effects of HA.

As acknowledged by the MRC framework [42–44] contextual aspects such as organizational structure, work climate, and staff turnover rates affect research use in practice. In our study, such aspects might have had an impact on how the occupational therapists in the intervention municipalities as a team adopted the standardized protocol. During the data collection, regular meetings were held with the occupational therapists in the intervention municipality with the aim to support fidelity to the intervention. Adopting and implementing research into practice is a complex process, requiring much effort and time to change routines, priorities and task distribution at the workplace [38]. Thus, considerable amounts of training and discussions are most often required to transfer research into practice contexts. At the onset of this study, the municipalities constituting the intervention group expressed a sincere intention to structure their practice to make it more efficient; such discussions had taken place before they were asked to participate in this study. However, despite the positive attitude towards change of practice there might have been some resistance and difficulties with the implementation among the individual occupational therapists. Time constraints and a large number of clients are common barriers for recruitment in this type of studies, and the fact that the standardized protocol was rather comprehensive might have had an impact on the fidelity. This might to some part contribute to explain the results showing only few differences in changes in outcomes over time between the two groups. Similar to other research in the field (see e.g. [23]), some components and procedures included in our intervention were also included in the ordinary practice applied in the control group, such as pre-HA assessments. This may thus cause some overlap between intervention and control group. Moreover, participants in both groups may have received other interventions than HA, such as assistive technology, in order to enhance independence or reduce decline. These might have blurred the investigated effects of the intervention so that differences between the two groups became too small to be significant. We can assume that the variations of such other interventions are random and similar in both groups in a trial like this, but this information is not available. That is, given the difficulties of standardizing the intervention and the number of interacting components, we see that implementing the same protocol for all people is a limitation. More flexibility regarding content and delivery than in the protocol applied, by including principles of client-centeredness, and goal-orientedness [41, 46] as recommended by Craig [44] and Greenhalgh [46], might have generated more significant effects. For example, using a range of standardized assessments chosen based on the specific needs and goals of the client would enable a more client-centered approach to HA. However, this needs to be investigated in further studies.

From a statistical point of view the lack of differences between the two groups may be due to a small sample size and thereby a lack of power to detect real differences. Prior to the study, power analyses were conducted indicating a sample size large enough to detect differences, however, as common in studies targeting older people and people with disabilities, high attrition rates resulted in a lower sample size than desired. Measures were undertaken to reach a sufficient sample size, such as a substantial prolongation of the data collection period in order to include more study participants. The group of people receiving HA in Sweden are increasingly facing health decline (see, for example [6, 8]), thus affecting their possibilities to participate in research studies requiring time and energy. In particular this is the case for people with cognitive decline [47] which constitute a considerable amount of those declining participation in our study. Since the data collection for this study comprised several different assessments at the same time point, this most probably contributed to the high attrition rate. Also, even though the outcome variables used in this study were selected based on prior research [25] and legislative frameworks, the measures selected for assessment and evaluation might not have been sensitive enough to detect any differences in changes between groups [48]. It should also be noted that we described the results in dichotomized terms (i.e. deterioration vs. no deterioration). Since the aim of this study was to gain an overall picture of the differences in changes over time between the intervention and ordinary practice using dichotomized data was considered sufficient. However, the use of non-dichotomized data could potentially have detected differences in changes in single items, but the general trend would most likely not have been affected.

## Conclusion

This study added to the professional judgements a standardized assessment and evaluation protocol for HA but this intervention did not show convincing effects. The reasons may be related to the complexity of HA and the structured assessment and evaluation protocol constituting the intervention in this study. The characteristics and motivation within the implementing municipality as well as the implementation process itself may also contribute to the challenges and thus lack of effect. This study was restricted to identifying trends in differences in changes between intervention and control sites over 1 year, but future studies applying more detailed data analysis to detect short-term differences would provide useful knowledge about the HA process regardless of the participants were in the control or the intervention group. A thorough process evaluation is necessary to gain in-depth knowledge about the reasons behind this lack of difference.

## Data Availability

Under the Statute (2003:615; SFS 2018:192) concerning the Ethical Review of Research Involving Humans and the Ethical Review Board approval (2012/566) the data is not publicly available.

## Abbreviations

ADL: Activities of Daily Living
ANOVA: Analysis of Variance
EQ VAS: EuroQoL Visual Analogue Scale
EQ-5D: EuroQoL-5Dimensions
HA: Housing Adaptation
MRC: Medical Research Council
SFS: Svensk Författningssamling (Swedish Governmental Regulations)
UIMH: Usability in My Home
